# Effects of Experimental Nitrogen and Phosphorus Addition on Litter Decomposition in an Old-Growth Tropical Forest

**DOI:** 10.1371/journal.pone.0084101

**Published:** 2013-12-31

**Authors:** Hao Chen, Shaofeng Dong, Lei Liu, Chuan Ma, Tao Zhang, Xiaomin Zhu, Jiangming Mo

**Affiliations:** 1 Key Laboratory of Vegetation Restoration and Management of Degraded Ecosystems, South China Botanical Garden, Chinese Academy of Sciences, Guangzhou, China; 2 State Key Laboratory of Urban and Regional Ecology, Research Center for Eco-Environmental Sciences, Chinese Academy of Sciences, Beijing, China; 3 Institute of Tropical Pratacultural Science, Zhanjiang Normal University, Zhanjiang, China; 4 University of Chinese Academy of Sciences, Beijing, China; Bangor University, United Kingdom

## Abstract

The responses of litter decomposition to nitrogen (N) and phosphorus (P) additions were examined in an old-growth tropical forest in southern China to test the following hypotheses: (1) N addition would decrease litter decomposition; (2) P addition would increase litter decomposition, and (3) P addition would mitigate the inhibitive effect of N addition. Two kinds of leaf litter, *Schima superba* Chardn. & Champ. (S.S.) and *Castanopsis chinensis* Hance (C.C.), were studied using the litterbag technique. Four treatments were conducted at the following levels: control, N-addition (150 kg N ha^−1^ yr^−1^), P-addition (150 kg P ha^−1^ yr^−1^) and NP-addition (150 kg N ha^−1^ yr^−1^ plus 150 kg P ha^−1^ yr^−1^). While N addition significantly decreased the decomposition of both litters, P addition significantly inhibited decomposition of C.C., but did not affect the decomposition of S.S. The negative effect of N addition on litter decomposition might be related to the high N-saturation in this old-growth tropical forest; however, the negative effect of P addition might be due to the suppression of “microbial P mining”. Significant interaction between N and P addition was found on litter decomposition, which was reflected by the less negative effect in NP-addition plots than those in N-addition plots. Our results suggest that P addition may also have negative effect on litter decomposition and that P addition would mitigate the negative effect of N deposition on litter decomposition in tropical forests.

## Introduction

Nutrient limitation, especially nitrogen (N) and phosphorus (P), to primary production and other ecological processes is widespread in terrestrial ecosystems [1], [2]. However, the differences are also obvious among systems. N is generally believed to be the main limiting nutrient in temperate forests [3], but the tropical forests more typically exhibit N-rich and P limitation [4]. While most studies have focused on the role of N on temperate ecosystems, nutrient limitation in tropical forest is not well studied.

Atmospheric deposition of N has increased drastically and is projected to increase further in tropical and subtropical regions in the coming decades [5], [6]. For example in China, the emissions of reactive N increased from 14 Tg N yr^−1^ in 1961 to 68 Tg N yr^−1^ in 2000 and is expected to reach 105 Tg N yr^−1^ in 2030 [7], leading to a high deposition of N (30–73 kg N ha^−1 ^yr^−1^) in some sub-tropical forests of southern China [8]. Increased N deposition has begun to alter nutrient status in these regions, which not only increases the N cycling rate [9], but also enhances P limitation in tropical forests. Some studies have demonstrated that elevated inputs of N to tropical forests would decrease P concentrations in soils and leaves [10], [11], suggesting the existence of a complex relationship between N deposition and P availability, and interactions that affect ecosystem processes in tropical forests.

Litter decomposition is a key ecosystem process that greatly influences the formation of soil organic matter, the release of nutrients for plants and microorganisms, and CO_2_ flux in forest ecosystems [12]–[14]. This process is also thought to be constrained by nutrient availability [15]. Previous studies of temperate forests have shown that N inputs increased [12], [16], decreased [17]–[20], or had no effect [21], [22] on litter decomposition. One of the reasons for these inconsistent responses may be related to different N levels in studied substrates or soils. Knorr et al. (2005) concluded that N addition may stimulate litter decomposition in N-limited forest, but inhibit litter decomposition in N- rich forest [23]. Therefore, in tropical forests, where soil N is relative rich, N input is expected to inhibit litter decomposition through aggravating C- limitation for microbial degradation, depressing microbial activity or soil fauna activity and more rapid formation of recalcitrant material[24]–[26]. However, direct measurements for this hypothesis are rare [24], [27].

In contrast to N, it is generally believed that P addition will increase litter decomposition in tropical forests due to low P levels [28]. However only five studies directly investigated this effect, and their results are inconsistent (some were positive [28], [29] and some were neutral [30]–[32]). In addition, direct evidence for the interaction of N deposition and P availability on litter decomposition is non- existent. Therefore, the nature and extent of the effects of N and P as well as their interactions on litter decomposition in tropical forests are still poorly understood.

To improve our understanding of the above questions, we established a full factorial N and P addition experiment in an old-growth tropical forest in the Dinghushan Biosphere Reserve (DHSBR), southern China. Earlier studies in this old-growth forest have shown that rather than retaining N there is a net loss of 8–16 kg N ha^−1^ yr^−1^ from the soil; these findings indicate this forest is completely N saturated from the chronic elevated N deposition [8], [33]. Furthermore, litter production and soil microbial biomass carbon in this forest significantly increased following addition of P, which suggests the forest is P-limited [25], [34]. Accordingly, we hypothesized that: (1) N addition would inhibit litter decomposition because of relative N-rich status in this forest; (2) P addition would increase litter decomposition because increasing P availability can meet the growth and maintenance requirements of decomposers in P-limited systems; (3) NP addition (interactive effect of N and P) would have less effect on litter decomposition compared to N or P addition alone, meaning that P addition would mitigate the inhibitive effect of N addition on litter decomposition.

## Materials and Methods

### Ethics Statement

No specific permits were required for the described field studies. This research station (DHSBR) belongs to South China Botanical Garden, Chinese Academy of Sciences, which also supported the study. We confirmed that the location is not privately owned. We also confirmed that the field studies did not involve endangered or protected species. Data will be made available upon request.

### Site Description

The study was conducted in DHSBR, an UNESCO/MAB site located in the central part of Guangdong Province, southeastern China (112°10' E, 23°10' N) with a total area of 1200 ha. Among the forest types at DHSBR is an old-growth monsoon evergreen broadleaf forest, which is about 250–300 m above sea level and occupies 20% of the reserve. This forest is typical of undisturbed forests of tropical China, having been protected by monks for more than 400 years and experiencing minimal direct human impacts [35].

The reserve has a typical monsoon and humid climate and is located in a subtropical/tropical moist forest life zone. The average annual precipitation of 1927 mm has a distinct seasonal pattern, with 75% occurring from March to August and only 6% falling from December to February [36]. The mean annual temperature is 21°C, with an average coldest (January) and warmest (July) temperature of 12.6°C and 28.0°C, respectively; annual mean relative humidity is 80% [36]. Inorganic N deposition measured in throughfall was 33 kg N ha^−1^yr^−1^, with an additional input as dissolved organic N at 15–20 kg N ha^−1^ yr^−1^ [33]. Soil type is lateritic red earth formed from sandstone [35]. The soil depth is more than 60 cm to the top of the C horizon [35]. The forest stands used in the experiment are situated on mountain slopes about 30°–35°. General soil properties are given in Table S1.

### Experimental Treatment

The experiment was a full factorial experimental design. This design included two main factors, N effects (two levels: without N addition and N addition with 150 kg N ha^−1^ yr^−1^) and P effects (two levels: without P addition and P addition with 150 kg P ha^−1^ yr^−1^). They constituted four (2×2) treatments in this experiment, including Control, N-addition (150 kg N ha^−1^ yr^−1^), P-addition (150 kg P ha^−1^ yr^−1^), and NP-addition (150 kg N ha^−1^ yr^−1^ plus 150 kg P ha^−1^ yr^−1^). Field plots were laid out randomly and also randomly selected to received these treatments (5 replicates per treatment). Plot size and fertilizer levels were similar to the experiment in Costa Rica by Cleveland and Townsend [37]. NH_4_NO_3_ and NaH_2_PO_4_ solutions, alone or combined, were sprayed once every other month to the forest floor with a backpack sprayer starting from February 2007 and continued through August 2008. Fertilizer was weighed and mixed with 5 L of water for each plot. Each control plot received 5 L of water without fertilizer.

### Litter Decomposition Experiment

Litter decomposition was determined by placing fresh litter in mesh bags in the plots. Two leaf litter types, *Schima superba* Chardn. & Champ. (S.S.) and *Castanopsis chinensis* Hance (C.C.), were chosen, since they are the dominant tree species and contribute most of the leaf litter to the study site. Leaf litter was collected using litter traps and nylon mesh placed on the forest floor under the trees in the study sites during May and June 2006 (before the start of N and P fertilization), the season of peak litterfall [38]. To obtain a uniform mixture, the litter of each species was mixed before filling the mesh bags. All litters were air-dried to a constant weight and six sub-samples (about 12.00 g per sub-sample) from each kind of litter were analyzed for initial N and P concentrations. N concentration was determined by the semimicro-Kjeldahl digestion method followed by the detection of ammonium with a Wescan ammonia analyzer, while total P concentration was analyzed colorimetrically after acidified ammonium persulfate digestion [39].

A total of 1000 litter bags were prepared from 25×25 cm polyvinyl screen with 0.5×0.5 mm mesh in the bottom and 2×2 mm in the top. Each bag was filled with 10.00 g airdried mass of litter. Only one litter type was put in each bag. At the end of February 2007, these litter bags were evenly distributed in each plot. Litter bags were randomly retrieved at about 3, 6, 9, 12, and 18 months after the start of the study. Five litter bags of each species (a total of 200 bags on each collection) were collected from each plot at each sample time. After collecting, we removed litter from litterbags and gently cleaned roots, soil and other extraneous materials. Leaf residues were oven dried at 45°C for 48 h and weighed. Litters from the last sampling date were measured for N and P concentrations using the method as described above.

### Soil Microbial Biomass and Community Structure

Soil sampling was conducted in August 2008. From each plot, 5 soil cores (2.5 cm inner diameter) were collected randomly from a 10-cm soil depth and combined to create one composite sample. Soil microbial biomass and community structure were characterized using phospholipid fatty acid (PLFA) analysis as described by Bossio and Scow [40]. The abundance of individual fatty acids was determined as nmol per g of dry soil and standard nomenclature was used [41]. Concentrations of each PLFA were calculated based on the 19∶0 internal standard concentrations. Frostegård and Bååth [42] chose a set of fatty acids to represent bacterial PLFAs, out of which i14∶0, 15∶0, i15∶0, a15∶0, i16∶0, 16∶1ω7c, 17∶0, a17∶0, i17∶0, cy17∶0, 18∶1ω7 and cy19∶0 were present in our samples. We calculated the sum of i14∶0, i15∶0, a15∶0, i16∶0, a17∶0 and i17∶0 as an indicator of gram-positive bacteria. Gram-negative bacteria were identified by the PLFAs: 16∶1ω7c, cy17∶0, 18∶1ω7 and cy19∶0 [43]. The fungi were identified by the PLFA 18∶2ω6,9c [44], and PLFAs 16∶1ω5c were used as a marker for arbuscular mycorrhizal fungi (AMF) [45]. The actinomycetes were identified by the PLFAs 10 Me 16∶0, 10 Me 17∶0 and 10 Me 18∶0 [46]. Other PLFAs such as 14∶0, 16∶0, 16∶1 2OH, 16∶1ω9c, 17∶1ω8c, 18∶1ω9c, 10 Me 19∶0, 18∶3ω6c and 20∶1ω9c were also used to analyze the composition of microbial community. The ratio of 18∶2ω6,9c to total bacterial PLFAs was used to estimate the ratio of fungal to bacterial biomass (F:B) in soils [42], [47].

### Data Analysis

The model for decomposition that we used was represented by the following equation [48]: y = e ^(−*k*t)^, where y is the fraction of mass remaining at a specific time t (years), “e” the base of natural logarithm, *k* the decomposition coefficient (year^−1^). Remaining nutrient content was calculated by multiplying the nutrient concentration by the mass remaining [31]. A simple T-test was used to determine the difference in initial litter chemical properties between two litter species. A two-way Analysis of Variance (ANOVA) with N and P as factors was performed to determine the effect of N and P addition on microbial characteristics. A three-way ANOVA with N, P and litter types as factors was performed to determine the effect of N and P addition and litter types on litter decomposition rate (*k*), nutrient remaining. To determine the effect of N and P addition on mass remaining across the time, repeated measures ANOVA with Turkey’s HSD test was performed using a mixed model in each litter type. All analyses were conducted in SAS software (SAS Institute Inc., Cary NC, USA). Statistically significant differences were set with *P* values <0.05 unless otherwise stated. Mean values ±1 standard errors were reported in the text.

## Results

### Initial Chemical Properties of Litter

Initial chemical composition was different between two litter types. S.S. leaf litter was higher than C.C. leaf litter in total N concentration, C/P ratio and N/P ratio. However, the C.C. leaf litter had a higher total P concentration and C/N ratio compared to that of S.S. leaf litter (Table 1).

**Table 1:** Initial properties of litter.

Litter types	TOC	TN	TP	C/N	C/P	N/P
	(mg g^−1^)	(mg g^−1^)	(mg g^−1^)			
S.S	445 (8) a	15.2 (0.3) a	0.56 (0.01) a	29.2 (0.9) a	802 (20) a	27.1 (8.5) a
C.C	434 (7) a	13.2 (1.0) b	0.79 (0.01) b	32.9 (0.3) b	551 (10) b	16.7 (2.7) b

S.S.: *Schima superba* Chardn. & Champ.; C.C.: *Castanopsis chinensis* Hance. Significant differences (p<0.05) among treatments are indicated by different letters. Error bars show SE (n = 6).

### Litter Mass Loss

**Figure 1:**
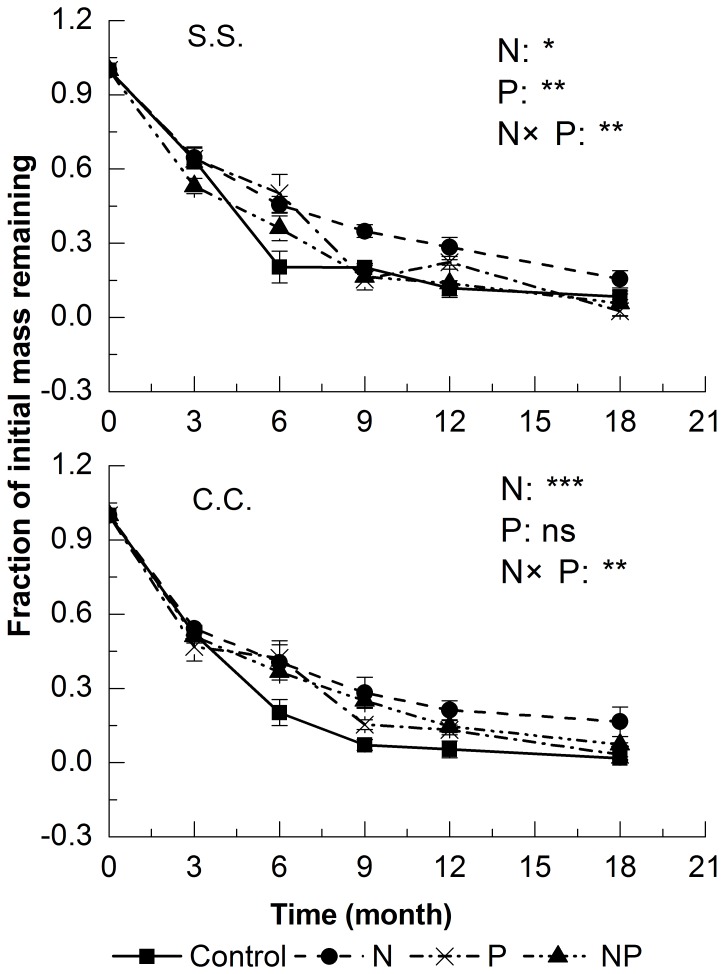
Mass loss of two decomposing leaf litters (S.S. and C.C.) in various nutrient addition treatments. The level of difference for the effect of N, P, and N and P interaction over the whole study period are indicated as ns : *P*>0.05; *: *P*<0.05; **: *P*<0.01; ***: *P*<0.001.

Litter decomposition rate varied depending on time and litter types (Fig. 1). The mass remaining in litter bags exponentially decreased with time and was characterized by an initial faster rate of disappearance, followed by a subsequent slower rate for all litter types. The regression equations describing decomposition rates over time were significant (*P*<0.05) for all litter types (coefficients of determination (R^2^) varied from 0.63 to 0.88, but most exceeded 0.75). Additionally, the two litter types differed significantly in decomposition rates. The decomposition rate of S.S. leaf litter (*k* = 3.38) was significantly faster than C.C. leaf litter (*k* = 2.16) in control plots (Table 2, *P* = 0.034).

**Table 2:** Mean decomposition rates (*k*) for S.S. and C.C. decomposed in the different nutrient addition treatments.

Treatments	S.S.		C.C.	
	*k*	*R^2^*	*k*	*R^2^*
Control	2.16 (0.25) a	0.63 (0.11)	3.38 (0.37) a	0.72 (0.25)
N	1.58 (0.38) b	0.88 (0.10)	1.65 (0.23) b	0.77 (0.23)
P	2.09 (0.14) a	0.79 (0.09)	2.37 (0.11) c	0.76 (0.10)
NP	2.23 (0.25) a	0.79 (0.01)	2.15 (0.29) bc	0.79 (0.22)

Decomposition rate (*k*) and coefficients of determination (*R^2^*) are based on a single negative exponential model.

Significant differences (p<0.05) among treatments are indicated by different letters. Error bars show SE (n = 5).

### Nutrient Addition Effects on Mass Loss

Nutrient additions had significant effects on litter decomposition rates, but the magnitude of these effects differed both between litter types and among fertilization treatments (Table 2). Additions of N significantly inhibited decomposition in both litter types (Table 2, *P* = 0.012 and 0.003 for S.S. and C.C., respectively). Decomposition coefficients (*k*) of S.S. and C.C. were 1.58 and 1.65 respectively after 18 months of N treatment, which was significantly lower than those in control plots (3.38 and 2.16 for S.S. and C.C., respectively). A repeated measures ANOVA with Turkey’s HSD showed that the effects of N addition on litter decomposition rates varied depending on decomposition time. For S.S. leaf litter, there were significant differences in decomposition rates between control and the N-addition treatment on the second, third and fourth sampling dates (Fig. 1, all *P*<0.05). Decomposition rates of C.C. leaf litter differed significantly between control and the N treatment (*P*<0.05) except for the first date of sampling (*P* = 0.150).

P addition had no significant effects on the decomposition rate of S.S. leaf litter (Table 2, *P* = 0.631), but significantly decreased the decomposition rate of C. C. leaf litter (Table 2, *P*<0.001). A repeated measures ANOVA with Turkey’s HSD showed that the negative effect in C.C. litter was significant in the second and fourth sampling dates after P treatment (Fig. 1, all *P*<0.05). Moreover, the degree of negative effects in P-addition plots was smaller than those in N-addition plots (Table 2, *k* = 2.77 and *k* = 1.65 for N and P addition in C.C. litter, respectively).

N and P addition showed significant interactions on the litter decomposition (Fig. 1, *P* = 0.002 and 0.001 for S.S. and C.C., respectively). The combined addition of both N and P seemed to mitigate the negative effect of N addition alone in both two litter types. The decomposition coefficient (*k*) in NP-addition plots was significantly higher than in N addition alone (*P*<0.001, n = 10). However, there was no significant difference between NP-addition and P-addition (*P* = 0.652, n = 10).

### Remaining Nutrient Content after 18 Months of Decomposition

**Figure 2:**
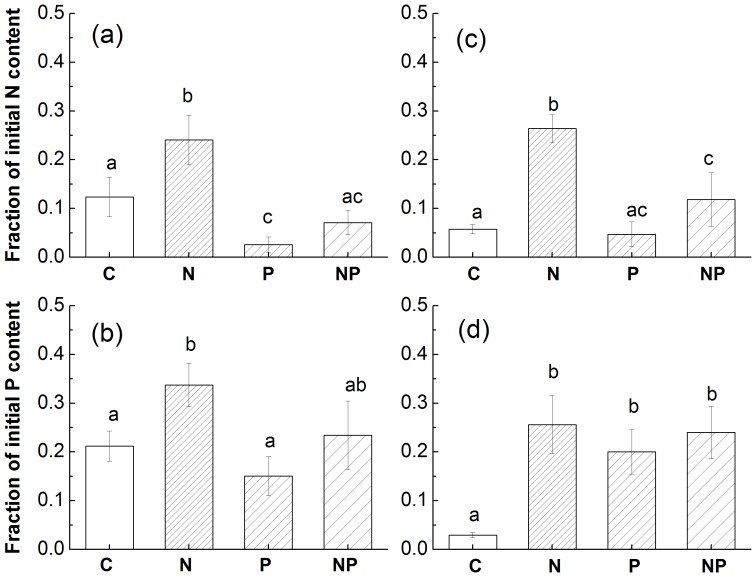
Comparison of remaining nutrient contents (fraction of initial nutrient content) among nutrient addition treatments in the late stage of decomposition. (a) and (b): S.S. leaf litter; (c) and (d): C.C. leaf litter. Significant differences (*P*<0.05) among treatments are indicated by different letters. Error bars show SE (n = 5).

After 18 months of decomposition, remaining N and P content were both significantly higher in N-addition plots than in control plots for both species (Fig. 2, all *P*<0.05). By contrast, the effects of P addition on the remaining nutrient content varied depending on species and nutrient (Fig. 2). In S.S. leaf litter, P addition significantly decreased N remaining (*P* = 0.025) and had no effect on P remaining (*P = *0.147) compared with control treatment. However in C.C. leaf litter remaining N content was not significantly different between P-addition and control (*P = *0.210), but the remaining P content was significantly higher in P-addition plots than in control plots (*P* = 0.032). A three-way ANOVA showed that the interaction between N and P treatment was not significant (*P* = 0.292 ).

### Soil Microbial Biomass and Community Structure

**Figure 3:**
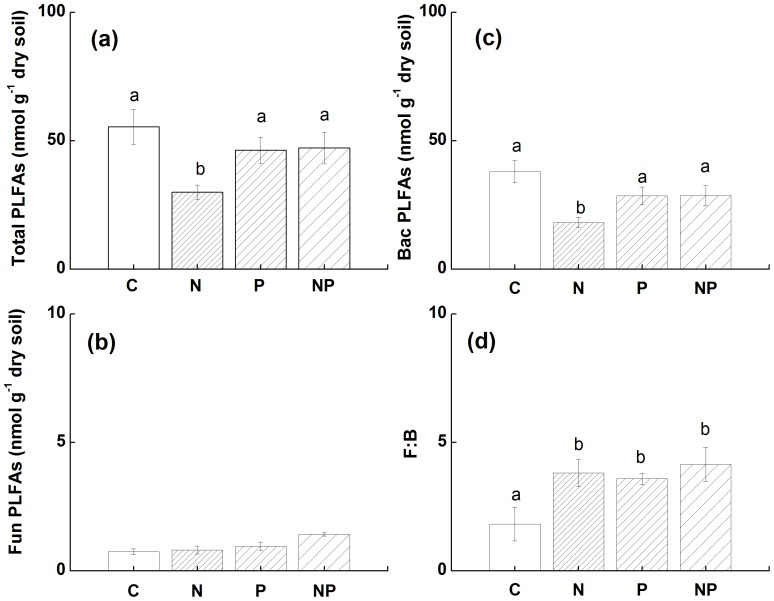
Comparisons of soil microbial PLFAs among nutrient addition treatments. Bac PLFAs: Bacterial PLFAs; Fun PLFAs: Fungal PLFAs; F:B: the ratio of fungal to bacterial PLFAs. Significant differences (*P*<0.05) among treatments are indicated by different letters. Error bars show SE (n = 5).

N addition significantly decreased soil microbial biomass. Total PLFAs and bacterial PLFAs in N- addition plots were significantly lower than those in control plots (Fig. 3, *P* = 0.009 and 0.008, respectively). However, there was no significant difference in fungal PLFAs between control and N addition (Fig. 3, *P* = 0.582). As a result, the ratio of fungal-to-bacterial PLFAs (F:B) was significantly higher in the N-addition plots (Fig. 3, *P* = 0.025). Although addition of P had no significant effect on soil microbial biomass (Fig. 3, *P* = 0.266), there was a significant interaction of N and P addition on total PLFAs (*P = *0.033).

**Figure 4:**
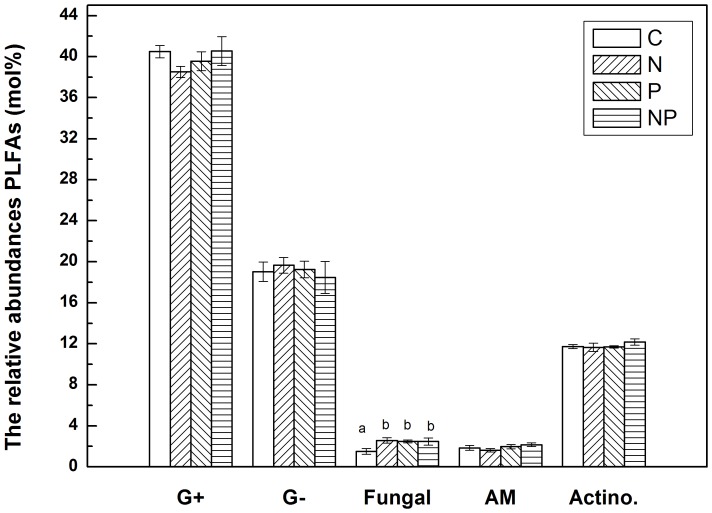
The relative abundances of the individual PLFAs (mol %) in soil samples. G^+^: the proportion of gram-positive bacterial PLFAs; G^−^: the proportion of gram-negative bacterial PLFAs; Fungi: the proportion of fungal PLFAs; AM: the proportion of AM fungal PLFAs; Actino.: the proportion of actinomycetes PLFAs. Significant differences (*P*<0.05) among treatments are indicated by different letters. Error bars show SE (n = 5).

Soil microbial community structure was changed after N and P addition. The relative abundances of fungal PLFAs in N-addition and P-addition plots were both significantly higher than those in control plots (Fig. 4, *P* = 0.029 and 0.046 for N and P addition, respectively). The interaction effect of N and P addition on relative abundances of fungal PLFAs was also statistically significant (*P* = 0.030).

## Discussion

As expected N addition decreased litter decomposition in the present study, which is consistent with previous studies in the same forest [24], [27]. N addition caused a decrease in litter decomposition in a number of ways. First, this mature forest is likely N saturated before N addition [33]. Second, a nutrient imbalance is likely to occur after N addition in this mature forest [24]. Third, higher N inputs retard the decomposition rate in older and more humified material in later stages of decomposition [24], [49]. Finally, N addition decreased the soil pH [27], microbial biomass [50] and the abundance and diversity of soil fauna [26]. In the present study, significant decrease in microbial biomass was also found after N addition (Fig. 3). In addition, N content in N-addition plots is significantly higher than those in controls in the late stage of decomposition (Fig. 2). This high N content after N addition generally indicates increased stabilization of plant residues due to the formation of stable organo-mineral complexes via condensation reactions between mineral N and N- rich organic matter [51], which in part support the third mechanism listed above.

The change in soil microbial community structure is also an important factor by which N addition regulates litter decomposition rate [52]. Recent studies suggest that fungi (e.g., white-rot basidiomycetes) would be repressed by N, leading to lower production of ligninase which is one reason for the N inhibition effect on decomposition [53], [54]. This hypothesis has been supported by work in temperate forests. For example, Fraterrigo et al. (2006) reported a negative relationship between soil fungal biomass and rates of N turnover in an Appalachian forest of North America [55]. Decreases in fungal biomarkers had also been found in soil of N-fertilized plots in Norway spruce forests [56]. However, here the change of microbial community structure was largely due to increases in relative abundance of fungal PLFAs, suggesting that soil microbial community structures may have different responses to N addition in tropical forests.

P addition also showed negative effects or no effect on litter decomposition in this study, which rejects our initial hypothesis. Three of five field experiments in tropical forests reported no response of litter decomposition to P addition [30]–[32], which are consistent with our observation on S.S. leaf litter. Two other studies found positive effects. Hobbie and Vitousek (2000) reported that elevated P (in litter) and elevated N+P (in soil) increased decomposition rates in a Hawaii tropical forest where P limits annual NPP. Kaspari et al. (2008) analyzed the relative effects of eight nutrient elements on litter decomposition and found that P availability is one of crucial factors to enhance litter decomposition in tropical forests. To our knowledge, no negative effects of P addition have been reported except for the present study.

We infer that this negative effect of P addition on litter decomposition in our study may be related to the suppression of “microbial P mining” proposed by Moorhead and Sinsabaugh [57]. They suggest that microbes decomposing organic matter in order to acquire P will be suppressed when there is sufficient P supply by increasing exogenous P addition. The suppression of “microbial N mining” was generally accepted as an explanation for the observed decline in litter decomposition with N addition. However, the suppression of “microbial P mining” is rarely proposed in P addition experiments [58]. In the present study, there are two ways this hypothesis is supported.

First, the remaining P content of decomposing C.C. leaf litter in the late stage of decomposition was higher in P-addition plots than those in controls (Fig. 2). A higher nutrient content of decomposing litter is generally related to microbial immobilization of nutrients [31]. This suggests that microbes more easily absorb P additions which may suppress P mineralization from the litter. Second, significant decreases in the production of soil phosphatase after P addition was also found (unpublished data), which is another evidence for the potential decease of P mining in this study. Previous work suggests that greater P availability could cause individual microbes use more labile C for growth instead of using it to produce P-mining enzymes such as phosphatase [59], [60].

Since N and P addition alone decreased litter decomposition, we expected the combined NP-addition would have a severe negative effect. However, there was a significant interaction between N and P additions in the present study. We found that the *k* value in NP-addition plots was significantly higher than those in N-addition plots, while there were not significant differences between P-addition and NP-addition (Table 2). This indicated that P addition mitigated the negative effects of N addition on litter decomposition. Several studies have reported that the addition of either N or P alone did not change litter mass loss, but combined N and P addition significantly accelerated litter mass loss [28], [32]. These studies implied that combined N and P addition may be more beneficial for litter decomposition compared to N addition alone, which partially supports our observation.

One possible explanation for this interaction is probably related to the neutralizing effects of N and P fertilization. Many studies have reported that N and P additions have opposing effects on ecosystem processes. For example, N addition can decrease soil pH, but P addition increases pH [27], [34]. N addition generally decreases microbial biomass [61], but P addition can increase microbial biomass [25]. Similar patterns were also found with other factors controlling decomposition, such as soil water content [34] and nutrient content in soil or litter layer [62]. As a result, P addition can partially mitigate the negative effect of N addition on litter decomposition.

In conclusion, a full factorial experiment was conducted to address the effects of N and P addition on the litter decomposition in an old-growth tropical forest. We have three important findings from this study: (1) N addition decreased the litter decomposition; (2) P addition inhibited litter decomposition, which may be due to the suppression of “microbial P mining”; (3) P addition may mitigate the inhibitive impacts of N addition. Our first finding indicates that N deposition may increase soil C sequestration in the tropical forests. The second finding provides a possible explanation for why P addition had no effect on litter decomposition in most previous studies. The authors of previous studies attributed findings of “no effect” to not using high enough nutrient doses, not treating plots long enough, or P not being a major factor affecting litter decomposition [28]; however they did not consider that high P additions may also have negative effects on litter decomposition in a P- limited tropical forest. Our third finding suggests that N and P have a complex interactive effect on litter decomposition such that increased P availability would mitigate the negative effect of N deposition on litter decomposition in tropical forests. However, the mechanisms underlying this are still far from clear and deserves further study.

## Supporting Information

Table S1 **Comparing of initial soil properties among four treatments.** Survey was conducted in February 2007 (before the start of N and P fertilization).
(DOC)
