# Supplementary material for: Effects of Experimental Nitrogen and Phosphorus Addition on Litter Decomposition in an Old-Growth Tropical Forest
Source: PLoS One. 2013 Dec 31;8(12):e84101. doi: 10.1371/journal.pone.0084101 (PMC3877223; doi:10.1371/journal.pone.0084101)
Supplement: Table S1 — Comparing of initial soil properties among four treatments. Survey was conducted in February 2007 (before the start of N and P fertilization). (DOC) [file pone.0084101.s001.doc]

**Table S1.** **Comparing of initial soil properties among four treatments.** Survey was conducted in February 2007 (before the start of N and P fertilization).

| Treatments | Control | + N | + P | + NP |
| --- | --- | --- | --- | --- |
| pH value (H_2_O) | 3.98 (0.02) | 3.99 (0.02) | 4.06 (0.02) | 3.07 (0.02) |
| Total N (mg g^-1^) | 1.99 (0.18) | 2.00 (0.14) | 2.16 (0.06) | 2.08 (0.11) |
| Organic matter (%) | 7.3 (0.8) | 7.7 (0.4) | 7.2 (0.5) | 7.9 (0.3) |
| C/N ratio | 21.0 (0.6) | 21.0 (0.5) | 20.0 (0.8) | 21.3 (0.6) |
| Total P (mg g^-1^) | 0.49 (0.03) | 0.44 (0.02) | 0.45(0.02) | 0.49 (0.01) |
| Available P (mg kg^-1^) | 2.2 (0.5) | 1.50 (0.4) | 1.9(0.2) | 2.1 (0.2) |
| Moisture (%) | 22.6 (1.1) | 23.5 (1.3) | 22.5 (2.0) | 22.4 (1.4) |
| Bulk density (g soil cm^-3^) | 1.0 (0.1) | 1.0 (0.1) | 1.0 (0.2) | 1.0 (0.1) |
| Microbial biomass C (mg kg^-1^) | 322 (36) | 313 (21) | 297 (26) | 362 (30) |

Note: No significant difference in soil properties among four treatments. Data were analyzed using one-way ANOVA with SNK test.
